# Regional cerebral effects of ketone body infusion with 3-hydroxybutyrate in humans: Reduced glucose uptake, unchanged oxygen consumption and increased blood flow by positron emission tomography. A randomized, controlled trial

**DOI:** 10.1371/journal.pone.0190556

**Published:** 2018-02-28

**Authors:** Mads Svart, Lars C. Gormsen, Jakob Hansen, Dora Zeidler, Michael Gejl, Kim Vang, Joel Aanerud, Niels Moeller

**Affiliations:** 1 Department of Internal Medicine and Endocrinology, Aarhus University Hospital, Aarhus, Denmark; 2 Department of Clinical Medicine, Aarhus University Hospital, Aarhus, Denmark; 3 Department of Nuclear Medicine and PET Center, Aarhus University Hospital, Aarhus, Denmark; 4 Section for Forensic Chemistry, Department of Forensic Medicine, Aarhus University Hospital, Aarhus, Denmark; 5 Centre of Functionally Integrative Neuroscience (CFIN) and MINDLab, Department of Clinical Medicine, Aarhus University Hospital, Aarhus, Denmark; 6 Institute of Biomedicine, Aarhus University, Aarhus, Denmark; 7 Department of Clinical Biochemistry, Aarhus University Hospital, Aarhus, Denmark; Swinburne University, AUSTRALIA

## Abstract

Ketone bodies are neuroprotective in neurological disorders such as epilepsy. We randomly studied nine healthy human subjects twice—with and without continuous infusion of 3-hydroxybutyrate–to define potential underlying mechanisms, assessed regionally (parietal, occipital, temporal, cortical grey, and frontal) by PET scan. During 3-hydroxybutyrate infusions concentrations increased to 5.5±0.4 mmol/l and cerebral glucose utilisation decreased 14%, oxygen consumption remained unchanged, and cerebral blood flow increased 30%. We conclude that acute 3-hydroxybutyrate infusion reduces cerebral glucose uptake and increases cerebral blood flow in all measured brain regions, without detectable effects on cerebral oxygen uptake though oxygen extraction decreased. Increased oxygen supply concomitant with unchanged oxygen utilisation may contribute to the neuroprotective effects of ketone bodies.

## Introduction

In evolutionary terms the ability to generate ketone bodies (3-hydroxybutyrate(3-OHB) and acetoacetate) has been fundamental for mankind surviving times of dwindling food supplies and ketosis induced by fasting, exercise, and—most recently–antidiabetic treatment with SGLT2-inhibitors may increase longevity in humans[1, 2]; it has also been reported that specific supplementation with 3-OHB increases lifespan in *C*. *Elegans* roundworms by 20%[3].

The brain relies on a constant rate of energy supply[4] and during metabolic stress eg. fasting, exercise, or severe disease, ketone bodies displace glucose as oxidative fuel[5, 6]. In hyperketonemic states 3-OHB is present in several fold higher concentration than acetoacetate[7] and there is evidence that 3-OHB promotes a more efficient ATP yield per oxygen molecule compared to lipid and carbohydrate fuels[8, 9]. Furthermore, 3-OHB has been reported to lower reactive oxygen species (ROS) dependent oxidative stress in the central nervous system (CNS)[10, 11], to inhibit histone deacetylase(HDAC) induced oxidative stress[12], and to promote the expression of brain derived neurotrophic factor [13]. A ketogenic diet has proven beneficial in the treatment of epilepsy and may also have favorable effects in the treatment of neurodegenerative diseases e.g. Alzheimer's and Parkinson's disease which are most prevalent in elderly people [14–17].

Our knowledge on the effects of ketone bodies on cerebral blood flow (CBF) and metabolism is limited, in paticular as regards acute hyperketonemia. Chronic hyperketonaemia in humans has been shown to induce a potent 50% reduction in global cerebral metabolic rate of glucose (CMR_glu_) after 4–6 weeks of fasting resulting in 3-OHB concentrations between 4–8 mmol/l [4, 18] and animal studies have suggested a 9% decline in glucose utlisation per 1 mmol increase in ketone bodies [19]. Acute studies in rats using the Kety-Schmidt technique have reported, that 3-OHB infusion increases global cerebral blood flow by 65% without altering oxygen or glucose metabolic rates[20]. One study in humans using the same technique with ^133^Xe dilution and internal jugular vein catheters found 40% increased CBF, 30% reduced CMR_glu_ and unaltered oxygen utilisation[21] after 3-OHB infusion in the fed state. Yet another study in humans also using internal jugular vein catheters showed unaltered CMR_glu_ and did not report measures of CBF or oxygen utilisation [22]; both studies used relatively low infusion rates with 3-OHB concentrations of ~ 2mmol/l and enrolled relatively young subjects with a mean age of 25 years. Human cerebral glucose utilisation decreases throughout life as well in human as in rats [23, 24]. Additionally, it is a prominent feature of mandis a prominent feature of upling our study design to studying elderly subjects. sed. not likely that it would have decresed Alzheimer’s disease patients and most neurogegenerative diseases affects elderly people[25]. Therefore, our study was designed to test whether an acute 4-h, high dose 3-OHB infusion affects CBF and oxygen and glucose utilisation in elderly humans, using PET and MRI with labeled [^15^O]H_2_O, [^15^O]O_2_ and [^18^F]FDG to assess regional CNS events.

## Material and methods

### Study participants

Nine healthy subjects were recruited after a screening procedure to ensure eligibility using the following inclusion and exclusion criteria. Inclusion criteria: Age 50–70 years, male or female, and body mass index (BMI) 20–30 kg/m^2.^ Exclusion criteria: Severe comorbidity, blood donation < 6 months, smooking, alcohol or drug abuse, clustrofobia, and participation in trials including radiation within the last 12 monts. All participants signed consent forms before enrolling. Screening and trial was carried out at the Aarhus University Hospital. The study was not blinded. Randomization sequence was generated at www.randomizer.org before the study was started.

The Danish Mid-Regional Ethics Committee approved the study (1-10-72-106-14), which was carried out in accordance with the declaration of Helsinki and registered at www.clinicaltrials.gov (ID number: NCT02357550).

### Study protocol

We used a randomized, controlled crossover design with two arms separated by at least 2 weeks: i) saline infusion (0.9%) (CTR) and ii) ketone body infusion with 3-OHB (75g/liter) (KET).

**Fig 1:**
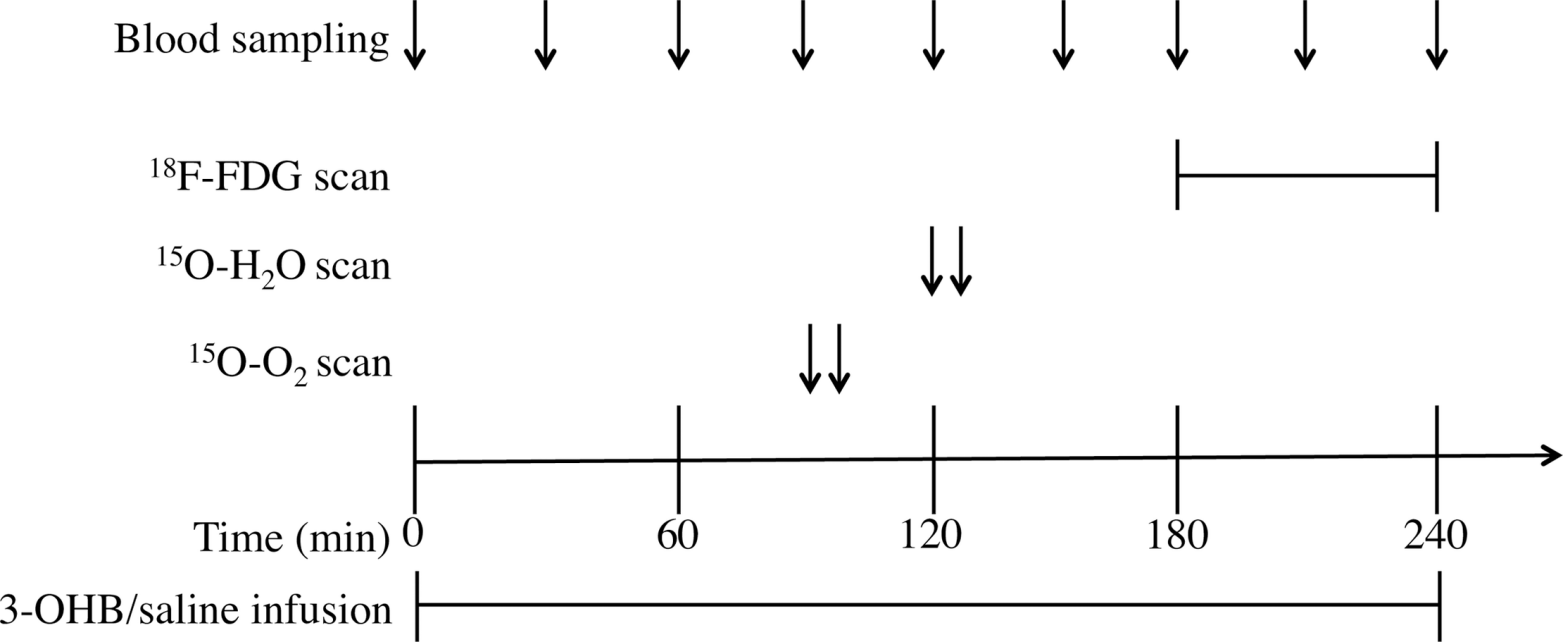
Flowchart. The flowchart illustrates the study design and shows the duration of 3-hydroxybuturate (3-OHB)/saline, and the timing of the three different scans and blood sampling.

Subjects were fasting overnight before each trial day. In the morning at 8 AM two catheters were placed in each antecubital vein; one for infusion and one for blood sampling, and an arterial cathether was placed in the radial artery. Infusions were started at 0 minutes and continued for 240 minutes. PET scans were performed at 120 min (1000 mBq ^15^O-O_2_), 150 min (500 mBq ^15^O-H_2_O), and 180 min (150 mBq ^18^F-FDG). During all scans the subjects were in the supine position with eyes closed. Blood samples were drawn hourly or ½hourly as indicated in Fig 1.

### 3-hydroxybuturate and saline

We infused DL-3-Hydroxybutyric acid, sodium salt (Gold Biotechnology, St Louis, MO) for four hours at a concentration of 75g 3-OHB/l and an infusion rate of 0.22 g/kg/hour. In the CTR isotonic saline was infused with a corresponding infusion rate.

### Plasma metabolites

Blood samples were stored at -20°C until analyzed. Serum FFA concentrations were quantified using enzymatic colorimetric method assay NEFA-HR (Wako Chemicals GmbH, Germany), serum lactate and glucose by immobilized enzyme biosensor technology (YSI 2300 model Stat Plus, Bie & Berntsen, Denmark). Serum concentrations of 3-OHB were measured using liquid chromatography tandem mass spectrometry[26].

Serum cortisol concentrations were determined by ELISA (DRG Cortisol Enzyme Immunoassay Kit, Germany), serum glucagon concentrations using a radioimmunoassay (EMD Millipore's Glucagon Radioimmunoassay Kit, Germany), and serum growth hormone concentrations using chemi-luminescence technology (IDS-iSYS hGH, Immunodiagnostic Systems Ltd, England).

### MR

A high-resolution 3D T1 weighted sequence was performed using a 32 channel head coil on a standard clinical 3T MAGNETOM Trio system (Siemens Healthcare, Erlangen, Germany) using a 3D T1 MPRAGE sequence with 176 slices, 1 x 1 x 1 voxel size, FOV 256 mm, TE = 3.7 ms, TR = 2420 ms, TI = 960 ms, flip angel = 9, iPAT = 2, 1 acquisition and with an acquisition time of 10:55. The MRI was done at the time of screening.

### PET scan protocols

All PET-images were obtained on a High Resolution Research Tomograph (Siemens Medical Solutions, Knoxville, TN, USA) in list mode. [^15^O]H_2_O-scans lasted five minutes and were reconstructed into 36 x 5 s, 6 x 10 s, and 3 x 20 s time frames. [^15^O]O_2_-scans lasted three minutes and were reconstructed into 12 x 5 s, 6 x 10 s, and 3 x 20 s time frames. [^18^F]FDG-scans lasted 60 minutes and were reconstructed into 6 x 10 s, 2 x 30 s, 4 x 60 s, 2 x 120 s, 2 x 300 s, and 4 x 600 s time frames. Arterial blood sampling was started simultaneously with tracer administration, and lasted for the duration of each scan. For [^15^O]H_2_O-scans, [^15^O]O_2_-scans, and the first 10 minutes of [^18^F]FDG-scans an Allogg automated blood sampler was used (Allogg AB, Mariefred, Sweden) to measure arterial radioactivity. During the last 50 minutes of [^18^F]FDG-scans, manual blood samples were taken at 10, 12, 17, 25, 35, 45, and 55 minutes, and activity counted on a Packard Cobra II system (Packard Instrument Company, Meriden, CT, USA). PET images were acquired in 1.22 mm steps isometric, with a field of view of 312 x 312 x 252 mm (x,y,z respectively).

PET images were coregistered to each individual's T1-weighted MRI. All MRI's were coregistered, by a combination of linear and non-linear coregistration, to a template consisting of 85 brains from young healthy people in ICBM152 space [27].

Co-registration was done with in-house software using an in-house MRI atlas in MNI-space, generic masks correspond to MNI coordinates. Transformations for PET-to-MRI and MRI-to-template were concatenated, and the resulting transformations were applied to dynamic PET-series to render them in common space. The automated coregistration algorithm extracted binary masks of cortical grey matter, which were multiplied with generic masks for each volume of interest.

Dynamic [^15^O]H_2_O- and [^15^O]O_2_-scans in common space were blurred to full-width half-maximum 7 mm. Parametric images of the unidirectional clearances (*K*_*1*_) of the two tracers were then calculated with the linearized two-compartement model [28], modified by Ohta *et al [29, 30]*. Cerebral metabolic rate of oxygen (CMR_o2_) was calculated by multiplying *K*_*1*_ voxel-maps of [^15^O]O_2_-scans with arterial oxygen concentration. The Lawson and Hanson non-negative least squares method was used to solve general least squares functions. We calculated parametric maps of CMR_glu_ using the Patlak method[31] with a lumped constant of 0.8[32]. Regions of interest were cortical grey matter, parietal, temporal, frontal, and occipital regions.

### Statistical analysis

Normal distribution of data was ensured by inspection of QQ-plots. The results are presented as means ± standard error of the mean (SEM), unless otherwise specified. The power calculation for the primary endpoint (CMR_glu_) as comparison of two paired means with significance level = 0.05, power = 0.8, difference of means = 3 umol/100 g brain tissue/min, and standard deviation of the mean = 2 umol/100 g brain tissue/min. This gave a sample size of N = 6. We included nine to ensure secondary outcomes as well. Secondary outcomes were CBF and CMR_o2_. Unequally distributed data were logarithmically transformed. Statistical association of the two trial days was tested using a paired two-tailed student’s t-test with eight degrees of freedom (t-test) and two-way repeated measure ANOVA analysis (ANOVA) when relevant. In case of significant main effect, pairwise comparison was made post hoc using a student’s paired, two-tailed t-test. A p-value <0.05 was considered significant. Graphs and statistical analysis were done using Stata 13 (College Station, Texas, USA) and Sigmaplot 11 (San Jose, California, USA).

## Results

**Fig 2:**
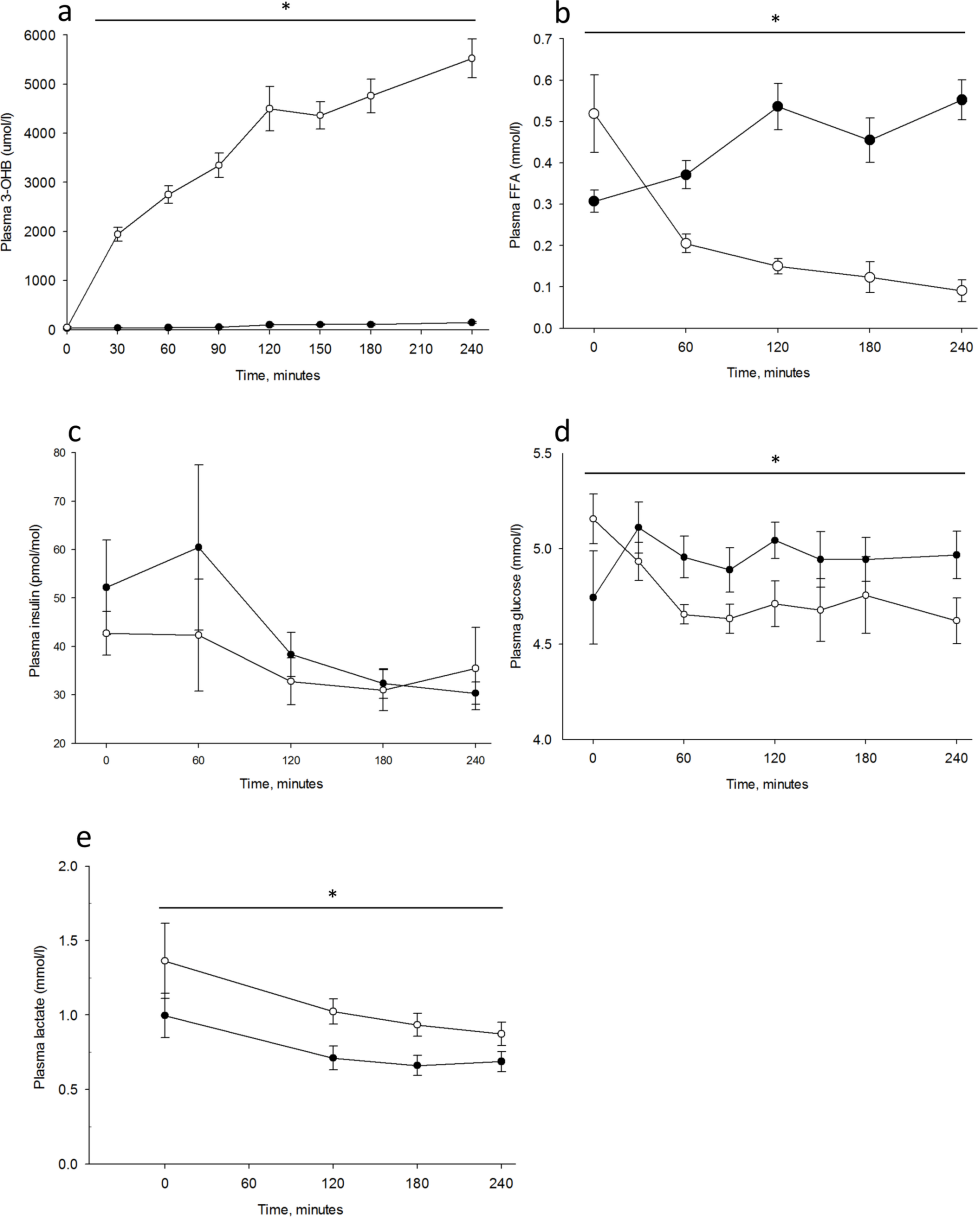
Metabolic parameters in the blood. Results are shown for n = 9 on Control day (CTR) and day with 3-hydroxybuturate (3-OHB) infusion (KET). Two-way repeated measures ANOVA analysis was used to test for differences between the two days. * = *p* <0.05. Open circles illustrate the mean value (±SE) on the KET day and closed circles illustrate the mean value (±SE) on the CTR day. Plasma 3-OHB (A) was measured every 30 minutes. Plasma free fatty acids (B) and insulin (C) was measured every hour. Plasma glucose (D) was measured every 30 minutes and lactate (E) at 0, 120, 180, and 240 minutes.

All nine subjects (four women), age 62 (range 56–69) and BMI 24±2 kg/m^2^ completed the two trial days and are included in the analyses. There were no adverse events. The intervention of infusing 3-OHB increased 3-OHB concentrations to 5.5±0.4 mmol/l during KET vs 0.2±0.02 during CTR, *p*<0.001 (main effect, ANOVA) (Fig 2A). Associated with the the increase in 3-OHB, the FFAs decreased significantly throughout the day during KET (Fig 2B).

Glucagon levels 57.8±5.5 pg/ml in KET vs 58.8±4.1 pg/ml in CTR and cortisol levels 119.7±21.7 ng/ml in KET vs 151.3±26.8 ng/ml in CTR were comparable, whereas growth hormone was significantly higher during KET (5.65±1.67 pmol/l) compared with CTR (1.82±0.80 pmol/l), p = 0.03 (t-test).

### Cerebral glucose metabolism decreases during hyperketonemia

**Fig 3:**
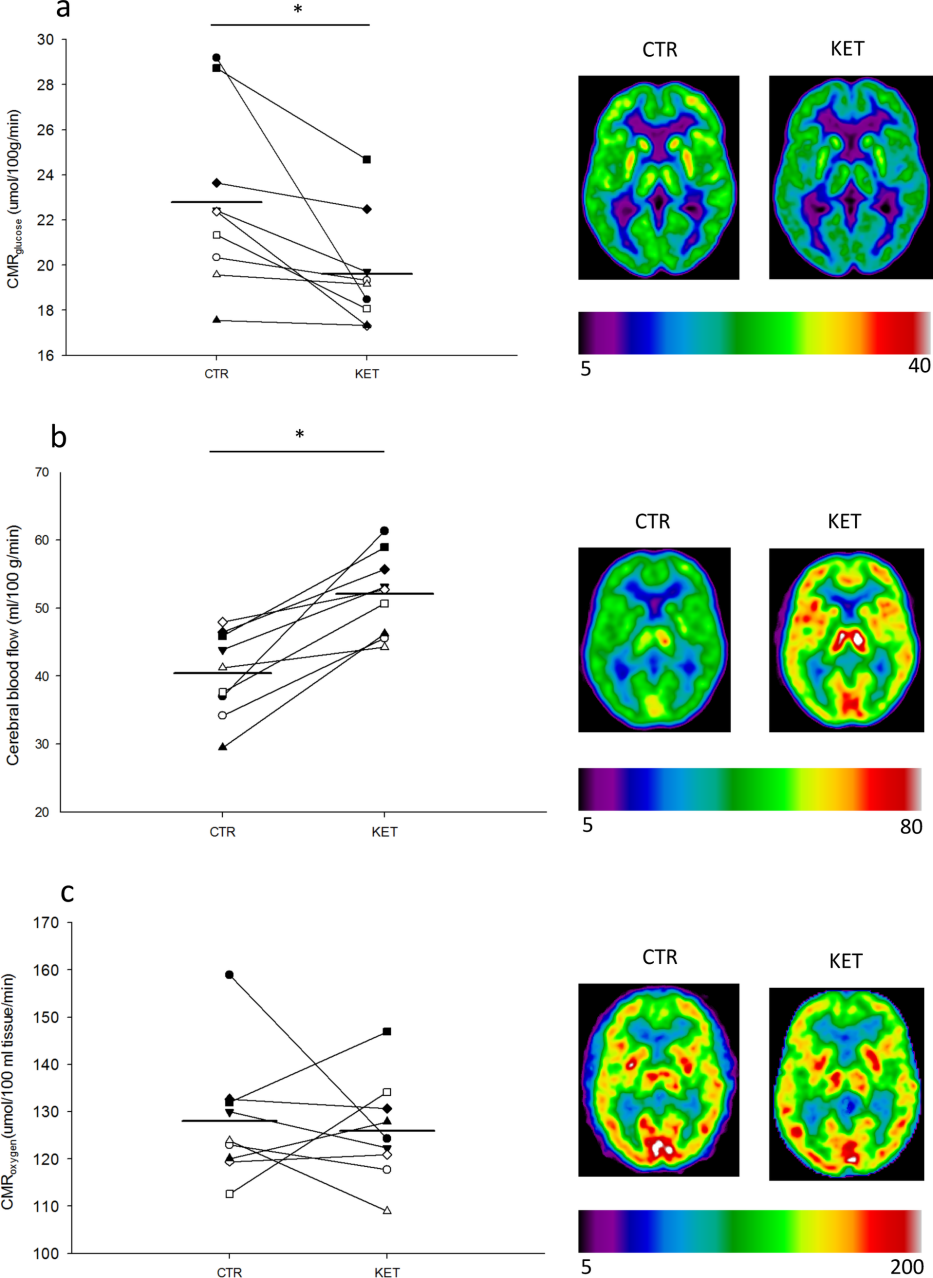
Individual PET-scan values. Results are shown for n = 9 on control day (CTR) and day with 3-hydroxybuturate (3-OHB) infusion (KET). Two-tailed T-test was used to test for difference between the two days. * = *p* <0.05. Data is presented as dot plots and each dot represents an individual value. The horizontal mean line indicates the mean value for each group. Cerebral metabolic rate for glucose (CMR_glucose_) (A) was measured at 180–240 minutes. Cerebral blood flow (B) was measured at 120–130 minutes. Cerebral metabolic rate for oxygen (CMR_o2_) (C) was measured at 90–100 minutes.

During acute hyperketonemia CMR_glu_ was reduced 14% in cortical grey matter; KET (19.6±0.8 mmol/100g/min) compared with CTR (22.8±1.3 mmol/100g/min), p = 0.02 (t-test) (Fig 3A). The same was found in frontal, temporal, parietal, and occipital regions, with the largest reduction in the frontal region Table 1. Plasma insulin concentrations declined comparably throughout both study days (Fig 2C), and plasma glucose concentrations remained slightly lower during KET compared with CTR, p<0.001 (main effect, ANOVA) (Fig 2D) at the time of FDG-scan for CMR_glu_. Lactate concentration was higher (p = 0.02 (main effect, ANOVA)) during KET compared with CTR (Fig 2E).

**Table 1:** Regional changes in cerebral metabolism during ketone body infusion.

CMR_glu_: umol/100g/min	CTR	KET	Difference of means (%-change from CTR)	P value
Frontal	24.3±1.4	20.7±0.9	3.5 (14.4)	**0.02**
Parietal	22.9±1.3	19.8±0.8	3.1 (13.5)	**0.02**
Temporal	20.6±1.2	17.7±0.7	2.9 (14.1)	**0.02**
Occipital	21.8±1.1	19.1±0.9	2.8 (12.8)	**0.03**
Cortical grey	22.8±1,3	19.6±0.8	3.2 (14.0)	**0.02**
**CBF:** umol/100g/min				
Frontal	41.6±2.3	54.2±2.2	12.6 (30.2)	**0.0007**
Parietal	40.9±2.1	52.5±2.0	11.5 (28.1)	**0.0007**
Temporal	36.5±1.9	47.4±1.9	11.0 (30.1)	**0.0004**
Occipital	42.2±2.0	52.3±1.6	10.1 (23.9)	**0.0007**
Cortical grey	40.4±2.1	52.0±2.0	11.6 (28.9)	**0.0006**
**CMR**_**o2**_: umol/100ml/min				
Frontal	127.6±5.0	125.6±3.1	2.0 (1.6)	0.74
Parietal	131.2±4.6	127.5±4.2	3.7 (2.8)	0.55
Temporal	117.1±3.8	117.1±3.4	0.0 (0)	0.99
Occipital	142.2±4.5	139.1±5.3	3.1 (2.2)	0.65
Cortical grey	128.1±4.4	126.0±3.6	2.1 (1.6)	0.71

In Table 1 we report absolute regional changes in PET-determined brain metabolism from the two experimental conditions KET-ketone body infusion and CTR-saline infusion; cerebral metabolic rate for glucose (CMR_glu_), cerebral blood flow (CBF), and cerebral metabolic rate for oxygen (CMR_o2_). A student’s paired two-tailed t-test was used to test for difference with eight degrees of freedom.

### Blood flow in the brain increases during hyperketonaemia

CBF increased 30% during acute hyperketonemia in cortical grey matter during KET (52.0±2.0 ml/100 g/min) compared with CTR (40.9±2.1 ml/100 g/min), p<0.001(t-test) (Fig 3B), the same pattern being observed in the frontal, temporal, parietal and occipital regions, Table 1. The frontal region accounting for the biggest increase. Heart rate increased during KET (14±2 beats per minute) compared with CTR (2±1 beats per minute), p<0.001 (t-test). We found no difference in mean arterial pressure 100±3 mmHg(CTR) vs 97 mmHg(KET), p = 0.24 (t-test). Arterial carbondioxide concentration and pH was unchanged in the two conditions and arterial oxygen partial pressure was slightly and significantly higher during CTR (10.5±1.1 kPa) compared with KET (9.5±1.2 kPa), p = 0.01 (t-test).

### Cerebral oxygen consumption is unaltered during hyperketonaemia

CMR_o2_ was unaltered during 3-OHB infusion CTR (128.1±4.4 umol/100 ml/min) vs KET (126.0±3.6 umol/100 ml/min), p = 0.71 (t-test) (Fig 3C) whereas the oxygen extraction was lower in KET (0.26±0.01 umol/partiel O2 pressure/ml) as compared with CTR (0.31±0.02 umol/partiel O2 pressure/ml), p = 0.04 (t-test).

## Discussion

Our study was designed to test whether an acute 4-h infusion with a high dose of 3-OHB affects CBF together with oxygen and glucose utilisation in humans using modern PET and MRI techniques. The main findings of this study using these techniques are that CMR_glu_ decreases 14% and CBF increases 30% under the influence of high 3-OHB concentrations. The 3-OHB concentrations in this study are equal to those seen after 4–8 weeks of fasting [18].

Our findings are in line with previous findings of substantially globally increased CBF, decreased CMR_glu_ and unaltered oxygen utilisation in rats [20] and in humans[21] measured with the Kety—Schmidt technique and internal jugular vein catheterisations. The human study also used PET-FDG to assess regional glucose metabolism, but did not report absolute changes in PET-determined glucose metabolism and only found minor regional differences of normalised rCMTglucose values. It should be noted that the study performed in humans used a lower infusion rate, resulting in concentrations of around 2 mmol/l of 3-OHB corresponding to 5–6 days of fasting[33]. Futhermore, the infusion was carried out for “3–3.5” h, “4–5 h” after a morning meal, implying that steady state conditions may not have been accomplished.

The 14% decrease in CMR_glu_ is somewhat lower than the 33% decrease found by *Hasselbalch et al*. It should be noted that circulating glucose levels were slightly, but significantly, elevated preceding the 3-OHB infusion. Insulin levels however were not significantly different, so this in all likelihood is an accidental finding; if anything increased glucose concentrations would tend to increase cerebral glucose uptake and metabolism as opposed to the 14% decrease observed after 3-OHB administration. The slight decrease in plasma glucose following infusion of 3-OHB that we observed, is not expected to play any significant role [34]. Age is thought to be important for the 3-OHB impact on CMR_glu_ as cerebral glucose consumption and metabolic flexibility decrease some 20% with age in humans[23] and some 30% in rats [24]. It has previously been found in rats, that one could expect a 9% decrease in CMR_glu_ pr 1 mmol/l increase in 3-OHB but again this finding was made in the young rats, and may not be relevant for older human beings[19]. The 14% reduction in CMR_glu_ may to large extent be explained by substrate competition between glucose and 3-OHB as originally described by *Randle et al*[35] in their seminal conceptual proposal which assigned important roles to both FFA and ketone bodies. It is unlikely that the recorded 0.1 mmol/l increase in lactate plays any significant role in this context[36].

CBF is tightly auto-regulated by oxygen supply and carbondioxide accumulation to secure constant conditions for the brain. As mentioned above *Hasselbalch et al* [21] reported a 40% increase in CBF in young human volunteers during short-term infusion of 3-OHB, a finding that could not be explained by alterations in CO_2_ tension or pH. This study was expanded in a rat model[20], but again without identification of a specific mechanism, despite thorough investigations of classic vascular and intracerebral regulators of CBF (pH, PaO2 and PaCO2). It remains possible that the ability of 3-OHB to restrict oxidative stress may induce vasodilation and increase blood flow[10, 12, 37]. Another plausible mechanism proposed by *Vlassenko et al* [38] links increased CBF with an increase in NADH/NAD+ as the result from 3-OHB conversion to acetoacetate.

The CMR_o2_ remained unchanged regardless of the two experimental conditions. As the CBF increased and the associated oxygen delivery was more or less unchanged, the oxygen extraction consequently decreased during hyperketonemia. This finding is supported by *Hasselbalck et al*, who did not find any altertions in oxygen consumption in the brain during hyperketonaemia. Studies in perfused rat hearts have indicated that addition of ketone bodies to the perfusate may increase hydraulic work efficiency per oxygene unit by 25%[39]. It has also been shown, that 3-OHB increases survival time to hypoxia markedly, up to four times[40]. A more recent study reported that 3-hydroxybutyrate infusion in ischemia induced by transient middle cerebral artery occlusion in mice, reduced infarct size, mediated by sirtuin 3, increased complex I activity in the mitochondria, and reduced ROS production [41]. In the mitochondria 3-OHB is also known to increase respiration resulting in increased brain derived neurotrophic factor expression, which in turn could also contribute to the neuro-beneficial effects of 3-OHB[42].

In conclusion we find that infusion with 3-OHB acutely increases CBF, and decreases CMR_glu_ in all measured regions of the brain. Increased oxygen supply and unchanged oxygen utilisation may—perhaps together with a favourable oxygen-ATP ratio and diminished oxidative stress—contribute to the neuroprotective effects of ketone bodies.

## Supporting information

S1 Fig (PDF)

S1 Table (PDF)

S1 Text (PDF)

S2 Text (PDF)
