# Supplementary figures and images for: Regional cerebral effects of ketone body infusion with 3-hydroxybutyrate in humans: Reduced glucose uptake, unchanged oxygen consumption and increased blood flow by positron emission tomography. A randomized, controlled trial
Source: PLoS One. 2018 Feb 28;13(2):e0190556. doi: 10.1371/journal.pone.0190556 (PMC5830038; doi:10.1371/journal.pone.0190556)

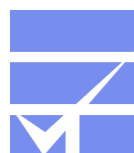

## CONSORT 2010 Flow Diagram

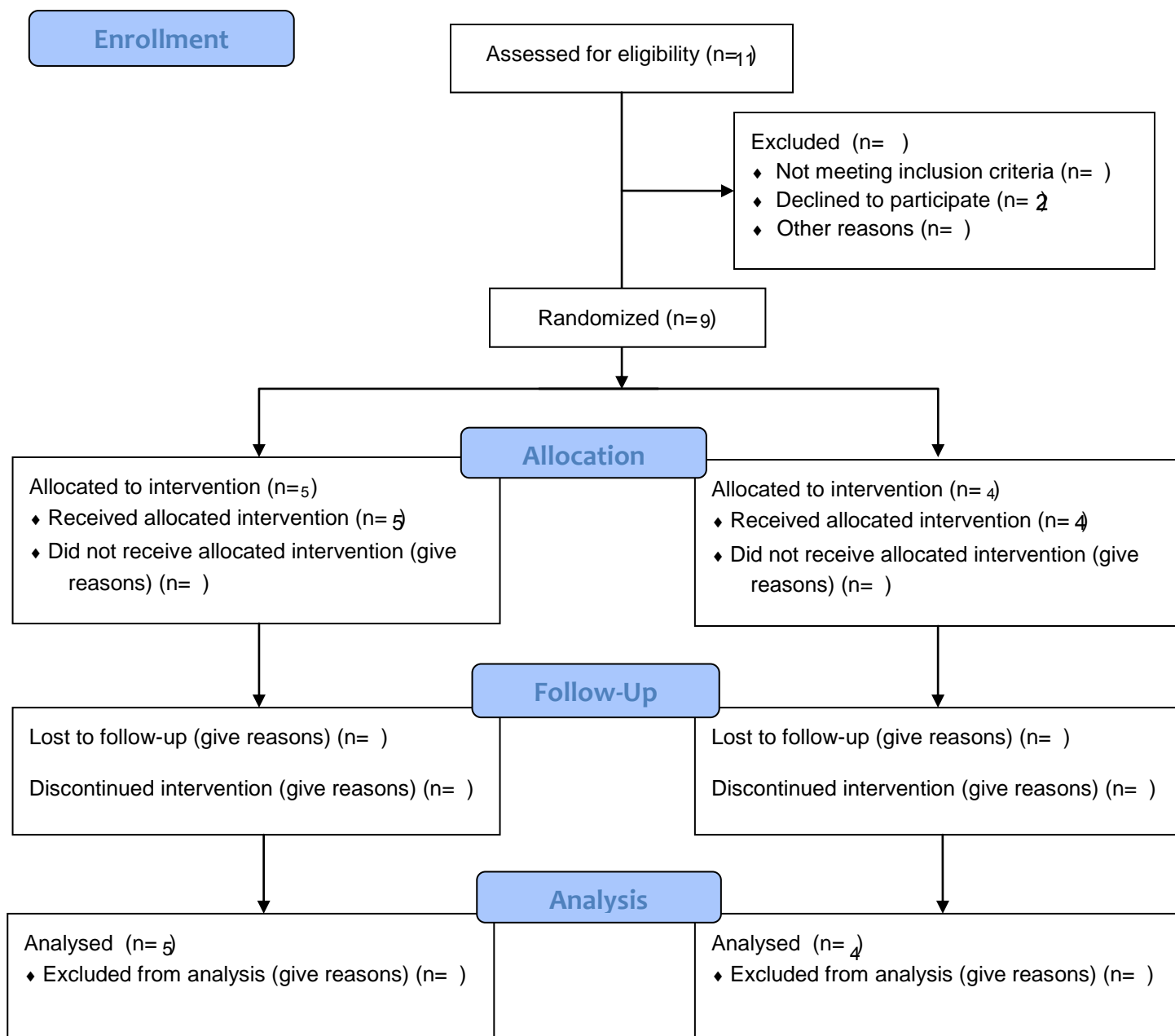

Supplement: S1 Fig — (PDF) [file pone.0190556.s001.pdf]
