# Supplementary material for: Regional cerebral effects of ketone body infusion with 3-hydroxybutyrate in humans: Reduced glucose uptake, unchanged oxygen consumption and increased blood flow by positron emission tomography. A randomized, controlled trial
Source: PLoS One. 2018 Feb 28;13(2):e0190556. doi: 10.1371/journal.pone.0190556 (PMC5830038; doi:10.1371/journal.pone.0190556)
Supplement: S1 Table — (PDF) [file pone.0190556.s002.pdf]

ID-ID	visit	CMRglu_Fro	CMRglu_pari	CMRglu_tem	CMRglu_occi
1	1	31,47	29,54	26,59	25,89
1	2	19,89	18,82	16,86	16,11
2	2	21,39	20,42	18,10	20,60
2	1	19,89	19,24	17,91	20,07
3	1	24,26	22,58	19,58	21,34
3	2	21,17	19,73	17,69	18,60
4	1	21,47	19,39	17,46	17,81
4	2	20,52	19,06	17,30	18,34
5	2	30,46	29,11	25,89	27,28
5	1	25,91	24,90	21,85	25,03
6	2	22,50	21,10	19,46	21,43
6	1	19,27	18,34	15,61	18,07
7	2	24,91	23,72	21,39	23,12
7	1	23,82	22,86	20,16	21,31
8	1	23,73	22,62	20,02	21,38
8	2	18,42	17,66	15,42	16,01
9	2	18,03	17,76	16,38	17,75
9	1	17,46	17,72	16,23	18,07

CMRglu_grey	Flow_Fronta	Flow_parieta	Flow_tempo	Flow_occipit	Flow_grey	CMRo2_Fron
29,19	38,71	37,54	33,51	36,53	36,99	160,92
18,47	65,36	61,70	55,64	57,76	61,32	123,73
20,34	34,32	35,10	30,47	37,98	34,18	119,11
19,32	47,15	46,12	40,19	48,30	45,55	117,07
22,43	46,87	44,77	38,21	42,35	43,85	130,09
19,71	55,89	52,91	49,05	51,99	53,13	123,56
19,57	43,91	41,21	36,62	40,70	41,19	126,44
19,15	46,50	44,59	39,94	44,04	44,24	111,93
28,74	47,07	46,37	42,16	47,24	45,87	129,26
24,68	61,02	59,74	53,66	59,46	58,93	143,65
21,33	37,49	37,85	34,17	43,23	37,58	111,85
18,06	52,73	51,49	45,14	52,18	50,65	132,13
23,64	47,50	46,76	42,65	48,77	46,42	136,54
22,49	58,18	55,88	51,27	54,49	55,67	132,17
22,37	49,51	48,43	42,84	50,49	47,93	117,63
17,30	54,64	52,79	47,78	54,48	52,69	124,24
17,55	29,11	30,30	27,44	32,73	29,47	116,40
17,32	46,40	47,03	44,01	48,24	46,24	122,06

CMRo2_pari	CMRo2_tem	CMRo2_occi	CMRo2_grey
161,60	144,70	172,26	158,97
125,51	116,57	136,74	124,27
126,30	114,85	141,66	122,98
117,64	113,21	127,23	117,71
136,65	110,44	149,76	129,95
122,86	111,41	135,68	122,27
124,23	112,73	133,79	123,89
108,71	97,87	118,21	108,90
136,64	122,35	147,30	132,01
152,23	134,48	167,33	146,95
114,02	104,43	126,08	112,58
136,48	124,89	151,57	134,12
135,56	116,69	142,14	132,69
133,28	120,61	137,89	130,65
123,00	112,56	130,37	119,41
120,72	113,03	123,28	120,84
122,70	114,98	136,60	120,08
130,25	121,77	153,84	127,84
