# Supplementary material for: Regional cerebral effects of ketone body infusion with 3-hydroxybutyrate in humans: Reduced glucose uptake, unchanged oxygen consumption and increased blood flow by positron emission tomography. A randomized, controlled trial
Source: PLoS One. 2018 Feb 28;13(2):e0190556. doi: 10.1371/journal.pone.0190556 (PMC5830038; doi:10.1371/journal.pone.0190556)
Supplement: S2 Text — (PDF) [file pone.0190556.s004.pdf]

Ketoners indflydelse på den cerebrale metabolisme af glukose: Et humant positron emissions tomografi (PET) studie

Projektansvarlige

Investigatorer:

Mads Svart, læge, ph.d-stud (primære investigator)
Lars Tolbod Poulsen, fysiker, ph.d.
Joel Aanerud, læge, ph.d.
Michael Geil, MD, PhD,
Lars C. Gormsen, overlæge, ph.d
Niels Møller, professor, dr. med.
.

Sponsor:

Nuklearmedicinsk afdeling & PET Center
Aarhus Universitetshospital
Nørrebrogade 44
DK-8000 Aarhus C
+4578463016
jf@ki.au.dk

Fysiske rammer

Nuklearmedicinsk afdeling & PET Center
Aarhus Universitetshospital

Medicinsk Forskningslaboratorium
Aarhus Universitetshospital

Kontakt

Mads Svart mvsv@clin.au.dk

Indhold

Ketoners indflydelse på den cerebrale metabolisme af glukose og fedtsyrer: Et humant positron emissions tomografi (PET) studie 3
Baggrund 3
Ketonstoffer 3
Relation til hjernens glukosemetabolisme og cerebralt blood flow 3
Dynamisk PET skanning 4
Formål 4
Endpoints 5
Design 5
Metoder 5
Inklusionskriterier raske forsøgspersoner: 6
Ekslusionskriterier raske forsøgspersoner: 6
Eksklusionsmuligheder under studiet 6
Forsøgsdag 1: 6
Forsøgsdag 2: 7
Statistik 9
Bivirkninger, risici og ulemper: 9
Sikkerhedsvurdering 10
Skriflig og mundtlig deltagerinformation 11
Rekruttering 12
Sted og tidsplan 12
Videnskabsetisk redegørelse 12
BUDGET – DRIFTSMIDLER 14
Økonomi 15
Kompensation for deltagere 15
Forsikring 15
Offentliggørelse af forsøgsresultater 15
Referencer / Bibliografi 16

Forsøgsprotokol

Ketoners indflydelse på den cerebrale metabolisme af glukose: Et humant positron emissions tomografi (PET) studie

Baggrund

Ketonstoffer

Hos mennesker med adgang til rigelige mængder kulhydrater dannes ketoner i leveren løbende i ganske små mængder, der kvantitativt udgør en forsvindende lille del af de cirkulerende næringsstoffer. Leverens produktion af ketonstoffer bliver til gengæld stærkt øget ved tilstande med manglende tilbud af kulhydrater, såsom under langvarig faste, i perioder med regulær sult, ved tilstande forbundet med hypoglykæmi, hos patienter med ukontrolleret diabetes, og såfremt man indtager kulhydratfattig kost (ketogen diæt). De væsentligste ketonstoffer hos mennesker er acetoacetat og beta-hydroxybutyrat, der produceres i leveren som følge af ophobning af intermediærmetabolitten acetyl-CoA, eksporteres derfra til blodet og bruges af perifært væv til energiproduktion (produktion af ATP) gennem Krebs' cyklus. De fleste organer kan forbrænde ketoner, men kvantitativt er hjernen og hjertet væsentligst. Forbrænding af ketoner kræver mindre iltforbrug per enhed ATP produceret sammenlignet med glukose, hvilket gør ketoner energieffektive. Ketonproduktion kan således tænkes at være et evolutionært fænomen, som sikrer et godt alternativ til glukose som energikilde. Herudover tyder adskillige nyere studier på, at ketoner har en række gavnlige effekter på flere sygdomsprocesser, bl.a. neurodegenerative sygdomme (Reger et al. 2004), kognitive forstyrrelser i relation til hypoglykæmi (Veneman et al. 1994) samt kardiovaskulær sygdom (Sharman et al. 2002). Det er derfor postuleret, at ketoner i fremtiden kan bruges i terapeutisk sammenhæng (Veech et al. 2001).

Relation til hjernens glukosemetabolisme og cerebralt blood flow

Under normale fysiologiske forhold med adgang til almindelig kulhydratrig kost forbrænder hjernen næsten udelukkende glukose. Dette ændres markant under langvarig faste, hvor de stigende mængder cirkulerende ketonstoffer bevirker et skifte i den cerebrale energiomsætning, der bliver langt mere afhængig af ketonstofforbrænding. Dette er vist i såvel flere dyrestudier og i enkelte små humane undersøgelser (Bentourkia et al. 2009, Pifferi et al. 2011, Blomqvist et al. 1995, Blomqvist et al. 2002). Sammenhængen mellem cirkulerende niveauer af ketoner og forbrændingen i hjernen er i disse studier stort set lineær. Hyperketonæmi påvirker tillige hjernens optag af alternative brændstoffer, herunder glukose, om end der er varierende opfattelser af, om ketoner medfører øget optag som postuleret af nogle grupper (Pifferi et al. 2011, Roy et al. 2013) eller om ketonerne omvendt begrænser optaget (LaManna et al. 2009). En nyere dansk kasuistik støtter sidstnævnte teori, idet en patient med behandlingsrefraktær epilepsi behandlet med langvarig ketogen diæt havde stærkt nedsat cerebralt optag af glukoseanalogen fluorodeoxyglucose- (FDG)

(Korsholm, Law 2013). Dette støttes af en metaanalyse, der har vist en omvendt lineær sammenhæng mellem niveauet af ketonstoffer i blodet og cerebral glukoseomsætning (Zhang et al. 2013). Hvorvidt der er forskel på effekten af hyperketonæmi afhængig af dennes varighed (kort- versus langvarig) er endnu ikke fuldt afklaret, og det har såvel aktuel klinisk relevans, som potentiel fremtidig behandlingsmæssig betydning at afklare dette nærmere.
Tidligere studier har således vist, at akut hyperketonæmi øger cerebralt blood flow uden samtidig stigning i global cerebral metabolisk aktivitet og dermed formentlig ligeledes uden stigende glukoseoptag (Hasselbalch et al. 1996, Linde et al. 2006). I modsætning hertil er den mere langvarige faste-inducerede hyperketonæmi associeret med uændret cerebralt blood flow (Redies et al. 1989, Hasselbalch et al. 1994).
Flere studier, såvel humane- som dyreeksperimentelle, har vist forskellige neuroprotektive egenskaber af ketoner. Dyremodeller af Alzheimers sygdom og Parkinsons sygdom tyder på beskyttende effekt af ketonstoffet beta-hydroxybutyrat, (Kashiwaya et al. 2000). Endvidere findes beskyttende effekt af hyperketonæmi i in vivo dyremodeller af henholdsvis traumatisk hjerneskade, iskæmisk hjerneskade og hjertestop, med reduceret skade på hjerne- og hjertevæv (Appelberg, Hovda & Prins 2009) (Tai 2008).
Endelig har dyrestudier vist, at ketoner regulerer flere signalveje, der synes at være associeret med beskyttelse mod oxidativt stress i såvel hjernen som i andre væv. (Shimazu et al. 2013)
Kun ganske få studier har undersøgt sammenhængen mellem induceret akut hyperketonæmi og cerebral glucosemetabolisme og af disse er kun et enkelt udført i mennesker (Hasselbalch et al. 1996). Der er derfor brug for flere undersøgelser af sammenhængen mellem akut hyperketonæmi og hjernens blodgennemstrømning og metabolisme.

Dynamisk PET skanning

Positron emissions tomografi (PET) er en nu veletableret teknik til ikke-invasiv påvisning og kvantitering af en række metaboliske processer *in vivo* hos mennesker. Ved PET-teknikken indgives en lille mængde af et radioaktivt mærket sporstof inkorporeret i et stof eller molekyle involveret i den fysiologiske/patologiske proces, man ønsker at undersøge. Ved en dynamisk PET skanning måles ændringer i den radioaktive aktivitet i et bestemt væv over tid, og sammenholdt med aktivitetskurven i plasma (inputfunktionen) kan optagelseshastigheden og omsætningshastigheden af sporstoffet i det pågældende væv kvantiteres.

^{18}F-fluorodeoxyglucose (FDG) bruges i klinisk diagnostik til påvisning af glukoseoptagende tumorer og inflammation, som ofte viser øget cellemetabolisme og dermed glukoseoptag. Disse skanninger er semikvantitative, idet man ikke er interesseret i den nøjagtige optagelseshastighed af glukose men mere i, hvorvidt et væv optager excessive mængder. Glukoseoptaget kan dog også nøjagtigt bestemmes ved dynamiske skanninger med efterfølgende matematisk bearbejdning af inputfunktionen og vævskurven.

Formål

Formålet med dette projekt er at undersøge effekten af ketonstoffer på hjernens optag og forbrænding af glukose.

Studiet har følgende hypoteser:
- At en akut øgning af ketonkoncentration i blodet hos raske mennesker vil føre til nedsat cerebralt glukoseoptag, samt til øget cerebralt blood flow.
- At en øgning af ketonkoncentration i blodet hos raske mennesker vil føre til nedsat iltforbrug i hjernen på grund af ketonstoffernes højere energieffektivitet.

Specifikke formål:
At kvantitere effekten af en kortvarig kontinuerlig ketoninfusion på hjernens optag af glukose, samt effekten på hjernens blodgennemstrømning og iltforbrug, vha. serielle dynamiske PET skanninger over hjernen med ^{15}O-vand, ^{15}O-O_2 og ^{18}F-FDG sporstoffer.

Endpoints

- Glucoseoptag i hjernen (^{18}F-FDG PET skanning)
- Glucoseforbrænding i hjernen (^{18}F-FDG PET skanning) – global & regional metabolic rate
- Blodgennemstrømning i hjernen (^{15}O-H_2O skanning)
- Hjernens iltforbrug (^{15}O-O_2 skanning)

Design

Studiet udføres som et randomiseret enkeltblindet overkrydsningsstudie med 10 raske forsøgspersoner. Hver deltager undergår to undersøgelsesdage – med og uden ketoninfusion – i tilfældig rækkefølge.

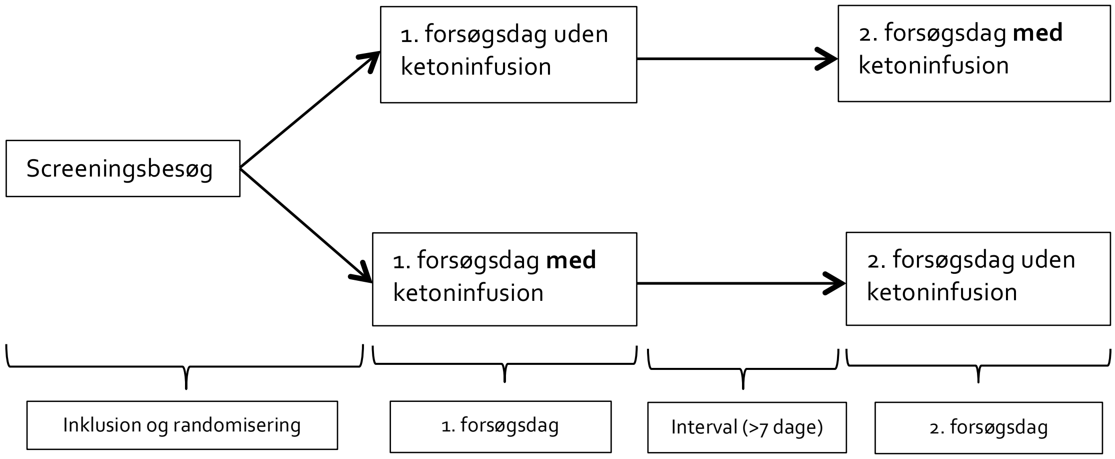

Metoder

Forsøgspersoner: Til studiet vil der blive rekrutteret raske forsøgspersoner. Rekrutteringen vil ske ved annoncering i lokale tidsskrifter samt på forsøgsperson.dk.

Inklusionskriterier raske forsøgspersoner:

- Alder: 50-70 år
- Mand eller kvinde-efter overgangsalder
- BMI: 20-30 kg/m^2

Eksklusionskriterier raske forsøgspersoner:

- Tegn på klinisk betydende hjerte-, lunge-, hjerne-, nyre-, lever-, endokrinologisk eller malign sygdom, evalueret ud fra anamnese, journaloplysninger samt basale blodprøver og EKG ved screeningsbesøg.
- Bloddonation i de sidste 6 måneder.
- Rygning.
- Kendt alkohol- eller stofmisbrug.
- Deltagelse i forskningsundersøgelser involverende ioniserende stråling inden for de sidste 12 måneder.
- Klaustrofobi.

Eksklusionsmuligheder under studiet

- såfremt forsøgsdeltageren skulle ønske det.
- såfremt der skulle opstå alvorlige eller intolerable bivirkninger.
- såfremt der skulle opstå et af ovennævnte ekslusionskriterier.

Screeningsbesøg: Ved dette besøg vil potentielle deltagere, som opfylder inklusionskriterier, informeres om projektet og få mulighed for at stille spørgsmål omkring deltagelsen og projektet. Følgende vil blive registreret:

- Anamnese mhp. eksklusionskriterier
- Vægt, højde og BMI
- Objektiv undersøgelse, inkl. puls, blodtryk og EKG (efter >5 min. i hvile)

Hvis forsøgsdeltageren vurderet ud fra ovenstående in- og ekslusionskriterier kan inkluderes, vil de blive tilbudt mulighed for at deltage i projektet. Der gives et par dages betænkningstid, hvorefter de kontaktes mhp. svar om deltagelse. Der tages i øvrigt en basal screeningsblodprøve (fastende) til bestemmelse af: Hæmoglobin, HbA1c, glukose, kolesterol, triglycerider, TSH, kreatinin, albumin, natrium, kalium, ALAT, ASAT, bilirubin og koagulationstal. Svar skal foreligge inden randomisering.

Forsøgsdag 1:

Deltagere møder fastende kl. 7.30 i PET Centeret, hvor der anlægges arterieadgang mhp. arterielle blodprøver under PET skanninger. Umiddelbart forinden udføres MR-skanning af hjernen med henblik på afklaring af de cerebrale anatomiske forhold.
Herefter udføres sekventielle dynamiske PET skanninger over hjernen, med bolus injektion eller inhalation af det relevante sporstof inden hver skanning. Sidste skanning afsluttes ca. kl. 14.

Efter sidste skanning tages en muskelbiopsi. Herefter fjernes arterie- og veneadgange og der serveres frokost.

Forsøgsdag 2:

Identisk med forsøgsdag 1, fraset at der indgives en kontinuerlig infusion af ketoner (natrium beta-hydroxybutyrat 0,24g/kg/min) under hele forløbet af forsøgsdagen, som afsluttes med en muskelbiopsi

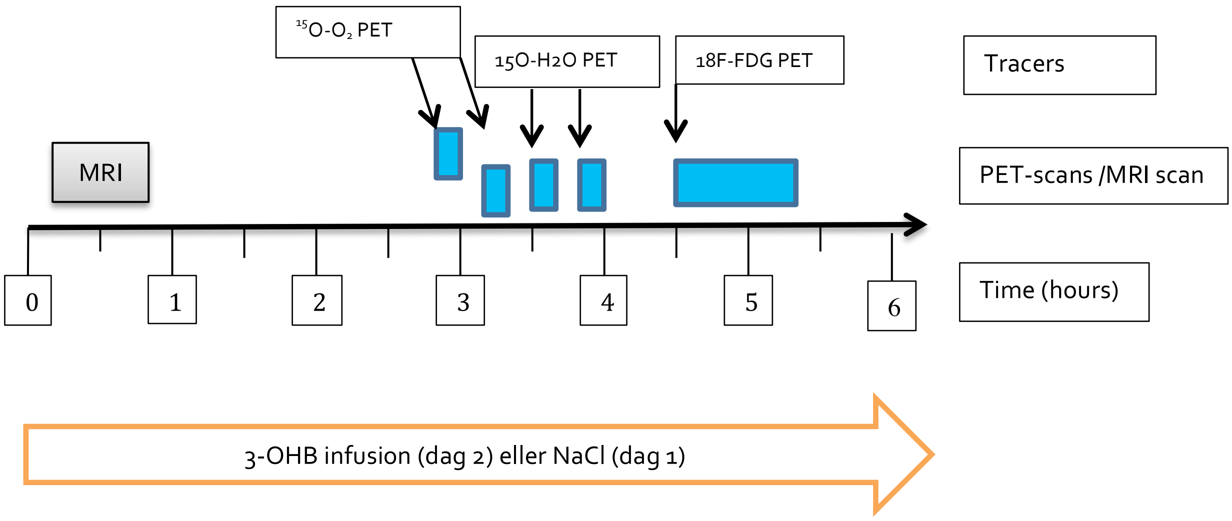

PET-skanninger: I løbet af hver forsøgsdag vil serielle dynamiske PET skanninger udføres i følgende rækkefølge:

1. **Iltmetabolisme-skan**
   Sporstof: $^{15}O-O_2$. Forsøgspersonen inhalerer luft der indeholder radioaktivt mærket O_2 (1000 MBq) og der optages dynamiske billeder af hjernen i 3 minutter. Undersøgelsen gentages efter 10 minutter med ny inhalation af radioaktivt mærket ilt til dobbeltbestemmelse af iltforbrug.
2. **Perfusions-skan**
   Sporstof: $^{15}O-H_2O$. Der gives en bolusinjektion af radioaktivit mærket vand (500 MBq) og der optages dynamiske billeder af hjernen i 3 minutter. Undersøgelsen gentages efter 10 minutter med ny injektion af radioaktivt mærket vand til dobbeltbestemmelse af cerebralt blood flow .
3. **Glucose-skan**

Sporstof: 18F-FDG. Der gives bolus injektion af traceren (150 MBq) og der optages dynamiske billeder af hjernen i 45 minutter.

MRI: Der optages magnetisk resonans billeder af hjernen med henblik på den efterfølgende anatomiske co-registrering og bearbejdning af af PET-billederne.

Blodprøver: Det samlede volumen for blodprøver udgør 100-250 ml per forsøgsdag.
Der tages en venøs blodprøve før påbegyndelse af de intravenøse infusioner og en anden i slutningen af forsøgsdagen efter skanningerne, hvorpå der måles følgende: glucose, acetoacetat, beta-hydroxybutyrat, insulin, c-peptid, glucagon, cortisol, adrenalin, leptin, ghrelin, væksthormon, frie fedtsyrer, triglycerider, cholesterol, lactat, pyruvat, urat, urea og elektrolytter.

I øvrigt tages flere mindre blodprøver i løbet af forsøgsdagen til bestemmelse af glykæmi og ketonæmi, samt under PET-skanninger til bestemmelse af sporstoffernes forsvindingskurve i plasma samt metabolitter til korrektion af inputfunktionen.

Muskelbiopsi: Et område på lateralsiden af det ene lår desinficeres med jodsprit og der lægges lokalbedøvelse med lidokain (10 mg/ml) svarende til hud, underhud og muskelfascie. Der laves en lille incision i huden og fascie med skalpel og der udtages med Bergstrøm biopsinål ca. 300 mg muskelvæv fra musculus vastus lateralis. Muskelbiopsien nedfryses i flydende kvælstof. Der tages i alt 1 biopsi.
Undersøgelser på udtaget væv: Insulin og protein signalering.

Forskningsbiobank: De ovennævnte blodprøver og muskelprøver opbevares i en forskningsbiobank og efter forsøgets afslutning opbevares de op til 15 år i anonymiseret form, hvorefter de destrueres. Formålet med forskningsbiobanken er, at udføre de biokemiske analyser, som er en del af denne protokol, samt at give mulighed for fremtidig brug i andre forskningsprojekter. Deltagere vil blive informeret om oprettelsen af biobanken og om muligheden for brug af deres materiale i fremtidige forskningsprojekter, samt om at de kan når som helst få alt deres opbevarede biologiske materiale destrueret, hvis de ønsker det. Eventuel fremtidig forskning på biologisk materiale fra forskningsbiobank skal godkendes af Videnskabsetisk Komité.

Patientdata: Ved indledende undersøgelse af personerne indsamles demografiske data og andre oplysninger: køn, alder, sygdomsdebut og varighed, medicin, sygdomsdisposition, øvrige sygdomme mm. Der oprettes en case report form (CRF) for hver deltager, heri sættes kildedata (originale dokumenter, der indeholder kliniske fund, observationer og øvrige oplysninger, der er nødvendige for at kunne rekonstruere et forsøg). Desuden vil antropometriske data som køn, alder, højde, vægt, BMI, puls og blodtryk blive indsat. Patientens journal vil ligeledes være at opfatte som kildedata, idet oplysninger om eventuelle eksklusionskriterier (betydende hjerte-, lunge-, hjerne-, nyre-, lever-, endokrinologisk eller malign sygdom) samt oplysninger om eventuelle tidligere undersøgelser med ioniserende stråling, vurderes forud for inklusion i studiet.

Databehandling: Data validitet sikres ved korrekturlæsning. Data arkiveres elektronisk bag adgangskode (manuelle data opbevares i aflåst lokale på Aarhus Universitetshospital) i anonymiseret form. Forsøgsdeltagerne registreres under et patientnummer og initialer og der oprettes en patientliste, hvor sammenhæng mellem navn, cpr og patientnummer registreres. Denne arkiveres separat. Gældende lov om behandling af personoplysninger vil blive overholdt.

Forsøgsdeltagere indvilger ved underskrift af samtykkeerklæringen samtidig i, at Videnskabsetisk komité, GCP-enheden og Datatilsynet kan få tilgang til forsøgsresultaterne. Studiet vil blive anmeldt til Datatilsynet.

Monitorering: Investigator tillader adgang til kildedata og kildedokumenter til medarbejdere fra de Videnskabsetiske Komitéer, Datatilsynet eller tilsvarende myndigheder i forbindelse med kontrol af data. Ved kildedokumenter forstås originale dokumenter med kliniske fund, observationer eller øvrige aktiviteter, der er nødvendige for rekonstruering og evaluering et forsøg. Herunder hører bl.a. patientjournaler/ udskrivningskort, laboratoriesvar, udskrifter fra apparatur og lignende. Den endelige præcisering af kildedata vil fremgå af et bilag til CRF. Deltagernes journaler forbliver på Aarhus Universitetshospital i den maksimale tid tilladt af Aarhus Universitetshospital. Investigator sørger for, at forsøgsdata fra CRF opbevares sikkert i minimum 5 år efter afslutning af forsøget.

Statistik

Resultaterne vil blive udtrykt som gennemsnit ± SE. Sammenligninger forsøgsdagene imellem vil blive foretaget med standard statistiske metoder (parret t-test eller lignende non-parametrisk test). P-værdi < 0,05 vil blive regnet for statistisk signifikant.
Styrkeberegning:
Styrkeberegninger vil ikke blive udført for alle effektmål, da vi ikke har relevante værdier for alle parametre, men baseres på glucoseforbrænding i hjernen, CMR_{glu}.
Tidligere studier har vist følgende værdier for mean CMR_{glc} (raske kontroller):
27,3 μmol/100g/min SD: +/- 1,9 N=6 (Grill et al. 1990)
30,7 μmol/100g/min SD: +/- 5,0 N=9 (Hasselbalch et al. 1994)
29,7 μmol/100g/min N=6 (Reivich et al. 1985)
22,6 μmol/100g/min SD: +/- 4,5 N=10 (Buchsbaum et al. 1989)
Vægtet mean: 27,23 μmol/100g/min
Spredningen estimeres ud fra (Reivich et al. 1985) ≈ 2,755 μmol/100g/min

Forventet ændring i CMR_{glc} ved keton infusion:
Hasselbalch har tidligere vist faldende CMR_{glc} (33,33%) ved akut induceret hyperketonæmi. (Hasselbalch et al. 1996)
Enkelte dyreeksperimentelle studier har dog vist stigende glucose optag i hjernen ved hyperketonæmi. (Roy et al. 2013, Pifferi et al. 2011)

I dette studie ønsker vi at kunne påvise en eventuel en *ændring* i hjernens glucoseforbrænding på 10% eller mere mellem placebo og intervention. (0,1 × 27,23 μmol/100g/min = 2,723 μmol/100g/min)

Signifikansen, ∝, sættes til 0,05 og β til 0,2 dvs. Styrke på 0,8. Der skal så rekrutteres 8,08 dvs. 9 deltagere i hver gruppe. Ved drop out vil forsøgsdeltageren søges erstattet fuldt.

Bivirkninger, risici og ulemper:

- Blodprøver: 100-250 ml per undersøgelsesdag. Anlæggelse af vene/arterie katetre kan medføre gener, og i nogle tilfælde hæmatom eller sjældnere infektion. Alle forsøgsdeltagere vil blive

informeret om at kontakte læge, såfremt der skulle opstå tegn på infektion (rødme, varme, hævelse, smerter).

- Beregning af strålebelastning:
  - 15O-H2O: 500 MBq x 0.0011 mSv/MBq = 0.55 mSv per injektion. Der udføres 2 skanninger per dag altså 1,1 mSv.
  - 15O-O2: 1000 MBq x 0,37 mSv/Gbq = 0,37 mSv per injektion. Der udføres 2 skanninger per dag altså 0,74 mSv.
  - 18F-FDG: 150 MBq x 0.019 mSv/MBq = 2,85 mSv per injektion (en pr dag)
  - Lavdosis CT mhp. attenuationskorrektion = 0,3 mSv per dag.
  - Samtidig vil vi ansøge videnskabsetisk Komité om at kunne gentage $^{15}O\text{-}H_2O$ injektion og $^{15}O\text{-}O_2$ inhalation og ledsagende billedoptagelse hvis den første optagelse fejler. Ekstra stråling ved behov for fornyet $^{15}O\text{-}H_2O$ + $^{15}O\text{-}O_2$= 0,55 + 0,37 mSv = 0,92 mSv.
  - Samlede strålebelastning = 4,99 mSv (5,91 mSv ved behov for ekstra ilt og vand optagelser) per undersøgelsesdag x 2 undersøgelsesdage = ~10 mSv (11,84 mSv ved behov for ekstra skan begge dage) svarende til lidt mere end 3 gange den baggrundsstråling man i Danmark normalt modtager i løbet af et år (ca. 3 mSv). Teoretisk beregning af den ekstra risiko ved deltagelse i forsøget vil ca. være en 0,05 % forøget risiko for pådragelse af en kræftsygdom i løbet af ens livstid, og en gennemsnitsdansker vil altså forøge sin risiko fra 25.00 % til 25,05 %.

- Bivirkninger til ketoninfusion: der er ikke beskrevet nogen bivirkninger til akut ketoninfusion som brugt i dette studies protokol. Det har tidligere været brugt på en lignende måde og dosisniveau i flere videnskabelige studier på mennesker uden rapporterede bivirkninger (Moller et al. 1990, Hasselbalch et al. 1996, Veneman et al. 1994, Pan et al. 2001).

Foranstaltninger ved bivirkninger: Deltagerne informeres om mulighed for hæmatom og infektion/phlebit i forbindelse med perifer kateteranlæggelse. Ved infektionstegn instrueres de i at søge læge.

De i forsøget anvendte tracere indgives i en mikroskopisk lille mængde. Anafylaksi forekommer ekstremt sjældent. Der er på PET centret de fornødne lægemidler mv. til at behandle en anafylaktisk reaktion. Der er altid en læge tilstede under skanningerne.

Sikkerhedsvurdering

- Deltagere vil få foretaget en generel helbredsscreening som forundersøgelse før deltagelse, med en almindelig objektiv undersøgelse inklusiv måling af vægt, højde, blodtryk og puls samt screeningsblodprøver og EKG.

- Laboratoriet har stor erfaring med alle de nævnte teknikker, og forsøgsdeltageren vil under hele forsøget være under observation af læge og bioanalytiker, der kan foretage almen vurdering af

deltageren samt foretage måling af vitale parametre (blodtryk, puls, blodsukker og bevidsthedsniveau) og intervenere om nødvendigt.

- Alvorlig hændelse defineres som: ethvert medicinsk tilfælde uanset dosis som resulterer i død, er livstruende, medfører hospitalisering eller forlængelse af eksisterende hospitalisering, resulterer i vedvarende eller betydelig anomali eller betydelig invaliditet/uarbejdsdygtighed eller giver en medfødt anomali/misdannelse .

- Hvis der indtræffer alvorlig hændelse/bivirkning rapporteres disse på et skema til indberetning af alvorlig hændelse/bivirkning set i forbindelse med kliniske forsøg og andre hændelser/bivirkninger noteres i CRF, der udfyldes for hver forsøgsperson.

- Ved alvorlige hændelser, som er en følge af projektet, vil den forsøgsansvarlige indberette de oplysninger, Videnskabsetisk Komité måtte anmode om.

- Hvis der optræder alvorlige hændelser/bivirkninger indsendes en liste til Videnskabsetisk Komité. Hertil sendes også en årlig sikkerhedsrapport.

- Registreringen af bivirkninger ophører, når forsøgsdagen slutter, idet det forventes, at de anvendte tracere er helt eller fortrinsvist udskilt af kroppen på dette tidspunkt. Kateter-komplikationer registreres fortløbende.

- Komplikationer forventes at kunne registreres i forbindelse med ovennævnte lægelige objektive observationer og behandles på forsøgslaboratoriet, idet forsøget udføres på et højt specialiseret sygehus.

- I tilfælde af alvorlig hændelse/bivirkning stoppes forsøget for den enkelte deltagere øjeblikkeligt og evt. påkrævet behandling institueres. Derefter vurderes det, om forsøget som sådant skal bringes til ophør.

- I tilfælde af hændelser/bivirkninger planlægges det at følge forsøgspersonen indtil symptomfrihed og/eller at tilstanden er stationær.

- Alle bivirkninger/hændelser vil endvidere fremgå af den afsluttende rapport.

- I tilfælde af hypoglykæmi vil der blive anvendt glucose 20% i.v. i relevant volumen og/eller glucagon i.v. Andre akutte medicinske tilstande vil ligeledes blive behandlet efter vanlige anerkendte instrukser.

Skriflig og mundtlig deltagerinformation

Den mundtlige og skriftlige information gives i overensstemmelse med Forskningsstyrelsens ”Forsøgspersoners rettigheder i et sundhedsvidenskabeligt forskningsprojekt”. Potentielle deltagere i undersøgelsen vil få udleveret skriftlig information om undersøgelsen og vil blive spurgt, om de ønsker at blive kontaktede med henblik på eventuel deltagelse. Nogle dage senere kontaktes den potentielle deltager af investigator for at afklare deres interesse i eventuelt at deltage. Ved positivt tilsagn aftales tid og sted for mundtlig information, som foregår ved ”screeningsbesøget” (forundersøgelse), hvor der også foretages en kort anamnese samt objektiv

lægelig undersøgelse mhp. eksklusionskriterier. Den mundtlige information gives i enerum af en læge, med mulighed for at medbringe en bisidder. Efterfølgende, og såfremt der ikke foreligger eksklusionskriterier, har den potentielle deltager mulighed for 2-3 dages betænkningstid, hvorefter de igen vil blive kontaktet af investigatoren med henblik på underskrift af den relevante samtykkeerklæring, såfremt de ønsker at deltage. Der skal foreligge fuldt informeret samtykke før screeningsblodprøver kan tages.

Der vil være mulighed for at stille spørgsmål, for at frasige sig ny viden om egen helbredstilstand og Den Centrale Videnskabsetiske Komité's informationspjece "Dine rettigheder som forsøgsperson i et biomedicinsk forskningsprojekt" udleveres.
Deltagerne vil blive informeret om, at de til enhver tid kan vælge at trække sig fra studiet uden forklaring og at dette ikke vil få konsekvenser for dem.

Rekruttering

Forsøgsdeltagerne rekrutteres gennem opslag i lokale aviser ~~og andre medier~~ eller på http://forsøgsperson.dk.

Sted og tidsplan

Studiet finder sted på Aarhus Universitetshospital: Medicinsk Endokrinologisk Afdeling (MEA) og Nuklearmedicinsk Afdeling & PET Center.

- Forventet start (inkl. rekruttering): april 2015.
- Forventet afsluttet: april 2016. Studiet er afsluttet, når den sidste deltager har gennemgået begge undersøgelsesdage.

Videnskabsetisk redegørelse

Man ved relativt lidt om ketoners metaboliske rolle hos mennesker, men den seneste forskning peger på, at ketonerne er kvantitativt og formentlig også kvalitativt langt vigtigere end hidtil anerkendt. Cirkulerende ketoners rolle som alternativ energikilde til hjernens energiforbrug ved for eksempel faste er velkendt, men det komplekse samspil mellem glukose- og ketonforbrug i andre væv herunder hjertet forstås ikke helt endnu.
Vi ved at neurodegenerative sygdomme som Alzheimers sygdom bl.a. er karakteriseret ved forstyrrelser i glukoseomsætningen i visse områder i hjernen.
En afklaring af samspillet mellem glukose og keton optagelse og omsætning i hjernen vil potentielt åbne mulighed for terapeutisk anvendelse af ketoner i behandling af cerebrale sygdomme i fremtiden, herunder neurodegenerative sygdomme.
Antallet af demensramte i DK anslås til 90.000 (2014, http://www.videnscenterfordemens.dk/viden-om-demens/tal-og-statistik) og på verdensplan 35.6 millioner med forventet fordobling frem mod 2030 (2012, http://www.who.int/mediacentre/news/releases/2012/dementia_20120411/en/).
En bedret forståelse af samspillet mellem keton- og glukoseomsætningen i hjernen vil også bidrage til forståelsen af virkningsmekanismer bag den neuroprotektive effekt af hyperketonæmi ved iskæmiske og traumatiske hjerneskader, som er vist i flere studier.

Resultaterne af disse studier af hhv. hjertets- og hjernens energiomsætning under hyperketonæmi vil potentielt kunne åbne flere muligheder for behandling af kardiologiske- og cerebrale sygdomme, enten ved at sænke patologisk øgede ketonniveauer, eller ved at administrere ketonstoffer terapeutisk. Risici forbundet med dette studie, primært bestrålingen fra de radioaktive sporstoffer, vurderes at være beskedne i forhold til den indsigt, studiet vil bringe. Studiet er designet med anvendelse af ikke-invasive metoder såsom PET til præcis bestemmelse af metaboliske processer in vivo, hvilket giver oplysninger som ellers er enten umulige eller mere risikable at undersøge ved andre metoder. Den samlede stråledosis på 9,96 mSv medfører, at projektet kategoriseres som gruppe IIb forsøg. Vi har taget højde for stråledosis ved kun at inkludere deltagere i alderen 50 år og over, og kan derfor i henhold til "Retningslinier vedrørende brug af ioniserende stråling i sundhedsvidenskabelige forsøg" øge stråledosis uden at udsætte vores forsøgsdeltagere for unødig risiko.

BUDGET – DRIFTSMIDLER

	PET CENTRET		
	Total pris	PET centrets bidrag	Netto Forskningspris
15O-H2O injektion (+ scan)	3.281,00	2460,75	820,25
18F-FDG (+scan)	14.000,00	11.500,00	3.500,00
MR-scanning			4.920,00
Brug af scannerrum (3 t)	9.843,00	13.237,50	2.460,75
Dynamiske blodprøver (15O) + O2	6.559,00	11.500,00	1.639,75
Dynamiske blodprøver (18F)	14.000,00	11.500,00	3.500,00
Opbevaring af data	2.000,00	1.500,00	500,00
Samlet pris per dag	81.333,00	60.999,75	**17.340,75**
X 20 undersøgelsesdage	1.626.660,00	1.219.995,00	**346.815,00**
Samlede udgifter PET			346.815,00
	**MEDICINSK FORSKNINGSLAB**		
	per enhed	Antal enheder	
Glucose analyse	10,00	40	**400,00**
Blod screenings analyse	200,00	1	**200,00**
Hormon analyser	20,00	13	**360,00**
Metabolitanalyser	20,00	13	**360,00**
Deltager godtgørelse	1.000,00	1	**1.000,00**
Samlet pris per dag			**2.320,00**
X 20 undersøgelsesdage			**46.400,00**
Arbejdsløn TAP	180.000,00	1	**180.000,00**
Samlede udgifter M-LAB			226.400,00
		**TOTALE UDGIFTER**	**573.215,00**
**FONDSMIDLER OPNÅET FRA ANDEN SIDE**			
		**STØTTE FRA ANDEN SIDE**	
**Driftsmidler samlet (DKK)**			**573.215,00**

Økonomi

Studiet er finansieret af fondsmidler fra KETO-gruppen. Såvel private som offentlige fonde vil blive søgt med henblik på opnåelse af driftsmidler.

Kompensation for deltagere

Deltagerne vil modtage et skattepligtigt beløb, til dækning af ulempegodtgørelse, på 1000 kr. pr forsøgsdag, som honoreres per påbegyndt forsøgsdag. Der gives kørselsgodtgørelse i dokumenteret omfang.

Forsikring

Deltagere er dækket i overensstemmelse med Lov om klage og erstatningsadgang inden for sundhedsvæsenet.

Offentliggørelse af forsøgsresultater

Studiets resultater tilstræbes publiceret i et internationalt peer-reviewed tidsskrift. Både positive og negative/inkonklusive resultater offentliggøres. Forfatterrækkefølge tentativt MVS, LPT, MG JA, LCG, NM .

Referencer / Bibliografi

1. Appelberg, K.S., Hovda, D.A. & Prins, M.L. 2009, "The effects of a ketogenic diet on behavioral outcome after controlled cortical impact injury in the juvenile and adult rat", *Journal of neurotrauma,* vol. 26, no. 4, pp. 497-506.

2. Bentourkia, M., Tremblay, S., Pifferi, F., Rousseau, J., Lecomte, R. & Cunnane, S. 2009, "PET study of 11C-acetoacetate kinetics in rat brain during dietary treatments affecting ketosis", *American journal of physiology.Endocrinology and metabolism,* vol. 296, no. 4, pp. E796-801.

3. Blomqvist, G., Alvarsson, M., Grill, V., Von Heijne, G., Ingvar, M., Thorell, J.O., Stone-Elander, S., Widen, L. & Ekberg, K. 2002, "Effect of acute hyperketonemia on the cerebral uptake of ketone bodies in nondiabetic subjects and IDDM patients", *American journal of physiology.Endocrinology and metabolism,* vol. 283, no. 1, pp. E20-8.

4. Blomqvist, G., Thorell, J.O., Ingvar, M., Grill, V., Widen, L. & Stone-Elander, S. 1995, "Use of R-beta-[1-11C]hydroxybutyrate in PET studies of regional cerebral uptake of ketone bodies in humans", *The American Journal of Physiology,* vol. 269, no. 5 Pt 1, pp. E948-59.

5. Buchsbaum, M.S., Gillin, J.C., Wu, J., Hazlett, E., Sicotte, N., Dupont, R.M. & Bunney, W.E.,Jr 1989, "Regional cerebral glucose metabolic rate in human sleep assessed by positron emission tomography", *Life Sciences,* vol. 45, no. 15, pp. 1349-1356.

6. Grill, V., Gutniak, M., Bjorkman, O., Lindqvist, M., Stone-Elander, S., Seitz, R.J., Blomqvist, G., Reichard, P. & Widen, L. 1990, "Cerebral blood flow and substrate utilization in insulin-treated diabetic subjects", *The American Journal of Physiology,* vol. 258, no. 5 Pt 1, pp. E813-20.

7. Hasselbalch, S.G., Knudsen, G.M., Jakobsen, J., Hageman, L.P., Holm, S. & Paulson, O.B. 1994, "Brain metabolism during short-term starvation in humans", *Journal of cerebral blood flow and metabolism : official journal of the International Society of Cerebral Blood Flow and Metabolism,* vol. 14, no. 1, pp. 125-131.

8. Hasselbalch, S.G., Madsen, P.L., Hageman, L.P., Olsen, K.S., Justesen, N., Holm, S. & Paulson, O.B. 1996, "Changes in cerebral blood flow and carbohydrate metabolism during acute hyperketonemia", *The American Journal of Physiology,* vol. 270, no. 5 Pt 1, pp. E746-51.

9. Kashiwaya, Y., Takeshima, T., Mori, N., Nakashima, K., Clarke, K. & Veech, R.L. 2000, "D-beta-hydroxybutyrate protects neurons in models of Alzheimer's and Parkinson's disease", *Proceedings of the National Academy of Sciences of the United States of America,* vol. 97, no. 10, pp. 5440-5444.

10. Korsholm, K. & Law, I. 2013, "Effects of a ketogenic diet on brain metabolism in epilepsy", *Clinical nuclear medicine,* vol. 38, no. 1, pp. 38-39.

11. LaManna, J.C., Salem, N., Puchowicz, M., Erokwu, B., Koppaka, S., Flask, C. & Lee, Z. 2009, "Ketones suppress brain glucose consumption", *Advances in Experimental Medicine and Biology,* vol. 645, pp. 301-306.

12. Linde, R., Hasselbalch, S.G., Topp, S., Paulson, O.B. & Madsen, P.L. 2006, "Global cerebral blood flow and metabolism during acute hyperketonemia in the awake and anesthetized rat", *Journal of cerebral blood flow and metabolism : official journal of the International Society of Cerebral Blood Flow and Metabolism,* vol. 26, no. 2, pp. 170-180.

13. Moller, N., Jorgensen, J.O., Moller, J., Orskov, L., Porksen, N., Alberti, K.G. & Schmitz, O. 1990, "Lack of effects of hyperglycemia on the disposal of 3-hydroxybutyrate in insulin-dependent diabetic patients", *Acta Endocrinologica,* vol. 123, no. 6, pp. 629-632.

14. Pan, J.W., Telang, F.W., Lee, J.H., de Graaf, R.A., Rothman, D.L., Stein, D.T. & Hetherington, H.P. 2001, "Measurement of beta-hydroxybutyrate in acute hyperketonemia in human brain", *Journal of neurochemistry,* vol. 79, no. 3, pp. 539-544.

15. Pifferi, F., Tremblay, S., Croteau, E., Fortier, M., Tremblay-Mercier, J., Lecomte, R. & Cunnane, S.C. 2011, "Mild experimental ketosis increases brain uptake of 11C-acetoacetate and 18F-fluorodeoxyglucose: a dual-tracer PET imaging study in rats", *Nutritional Neuroscience,* vol. 14, no. 2, pp. 51-58.

16. Redies, C., Hoffer, L.J., Beil, C., Marliss, E.B., Evans, A.C., Lariviere, F., Marrett, S., Meyer, E., Diksic, M. & Gjedde, A. 1989, "Generalized decrease in brain glucose metabolism during fasting in humans studied by PET", *The American Journal of Physiology,* vol. 256, no. 6 Pt 1, pp. E805-10.

17. Reger, M.A., Henderson, S.T., Hale, C., Cholerton, B., Baker, L.D., Watson, G.S., Hyde, K., Chapman, D. & Craft, S. 2004, "Effects of beta-hydroxybutyrate on cognition in memory-impaired adults", *Neurobiology of aging,* vol. 25, no. 3, pp. 311-314.

18. Reivich, M., Alavi, A., Wolf, A., Fowler, J., Russell, J., Arnett, C., MacGregor, R.R., Shiue, C.Y., Atkins, H. & Anand, A. 1985, "Glucose metabolic rate kinetic model parameter determination in humans: the lumped constants and rate constants for [18F]fluorodeoxyglucose and [11C]deoxyglucose", *Journal of cerebral blood flow and metabolism : official journal of the International Society of Cerebral Blood Flow and Metabolism,* vol. 5, no. 2, pp. 179-192.

19. Roy, M., Nugent, S., Tremblay, S., Descoteaux, M., Beaudoin, J.F., Tremblay, L., Lecomte, R. & Cunnane, S.C. 2013, "A Dual Tracer PET-MRI Protocol for the Quantitative Measure of Regional Brain Energy Substrates Uptake in the Rat", *Journal of visualized experiments : JoVE,* vol. (82). doi, no. 82, pp. 10.3791/50761.

20. Sharman, M.J., Kraemer, W.J., Love, D.M., Avery, N.G., Gomez, A.L., Scheett, T.P. & Volek, J.S. 2002, "A ketogenic diet favorably affects serum biomarkers for cardiovascular disease in normal-weight men", *The Journal of nutrition,* vol. 132, no. 7, pp. 1879-1885.

21. Shimazu, T., Hirschey, M.D., Newman, J., He, W., Shirakawa, K., Le Moan, N., Grueter, C.A., Lim, H., Saunders, L.R., Stevens, R.D., Newgard, C.B., Farese, R.V.,Jr, de Cabo, R., Ulrich, S., Akassoglou, K. & Verdin, E. 2013, "Suppression of oxidative stress by beta-hydroxybutyrate, an endogenous histone deacetylase inhibitor", *Science (New York, N.Y.),* vol. 339, no. 6116, pp. 211-214.

22. Veech, R.L., Chance, B., Kashiwaya, Y., Lardy, H.A. & Cahill, G.F.,Jr 2001, "Ketone bodies, potential therapeutic uses", *IUBMB life,* vol. 51, no. 4, pp. 241-247.

23. Veneman, T., Mitrakou, A., Mokan, M., Cryer, P. & Gerich, J. 1994, "Effect of hyperketonemia and hyperlacticacidemia on symptoms, cognitive dysfunction, and counterregulatory hormone responses during hypoglycemia in normal humans", *Diabetes,* vol. 43, no. 11, pp. 1311-1317.

24. Zhang, J., Cao, Q., Li, S., Lu, X., Zhao, Y., Guan, J.S., Chen, J.C., Wu, Q. & Chen, G.Q. 2013, "3-Hydroxybutyrate methyl ester as a potential drug against Alzheimer's disease via mitochondria protection mechanism", *Biomaterials,* vol. 34, no. 30, pp. 7552-7562.
